# Distinct effects of social motivation on face evaluations in adolescents with and without autism

**DOI:** 10.1038/s41598-018-28514-7

**Published:** 2018-07-13

**Authors:** Lou Safra, Christina Ioannou, Frédérique Amsellem, Richard Delorme, Coralie Chevallier

**Affiliations:** 1 Laboratoire de Neurosciences Cognitives, Inserm unit 960, Département d’Etudes Cognitives, Ecole Normale Supérieure, PSL Research University, Paris, 75005 France; 2 Service de Psychiatrie de l’Enfant et de l’Adolescent, Hôpital Universitaire Robert Debré, Paris, 75019 France; 3 Génétique Humaine et Fonction Cognitive, Institut Pasteur, Paris, 75015 France

## Abstract

Individual differences in social motivation have an influence on many behaviours in both clinical and non-clinical populations. As such, social motivation has been identified as a biological trait that is particularly well-suited for dimensional approaches cutting across neuropsychological conditions. In the present paper, we tested whether social motivation had a similar impact in the general population and in a neuropsychological condition characterized by diminished social motivation: Autism Spectrum Disorders (ASD). More precisely, we evaluated the effect of social motivation on face evaluations in 20 adolescents with ASD and 20 matched controls using avatars parametrically varying in dominance and trustworthiness. In line with previous research, we found in the control group that participants with higher levels of social motivation relied more on perceived trustworthiness when producing likeability judgments. However, this pattern was not found in the ASD group. Social motivation thus appears to have a different effect in ASD and control populations, which raises questions about the relevance of subclinical or non-clinical populations to understand ASD.

## Introduction

Compared to many other animals, humans stand out when it comes to the variety of social interactions they pursue and the importance of social activities in their ecological niche^1^. The willingness to be included in social interactions and the propensity to preferentially attend to the social world is present early on in development and remains a driving force throughout the lifespan^2–4^. Social stimuli such as faces and speech are granted special attention from birth^4,5^ and interactive activities are consistently favored over solitary ones by children as young as three^6^. In adults, social cues receive attentional priority^7–9^ and positive social feedback reinforces learning^10–12^. Yet, individuals vary in the degree to which they are socially motivated^13–18^ and atypicalities in social motivation are found in many clinical conditions^17,18^. Atypical social motivation is indeed an important characteristic of multiple psychiatric conditions, including Autism Spectrum Disorders (ASD)^19–22^, anorexia nervosa, schizophrenia and major depressive disorder^23,24^. Attention to social stimuli is also thought to provide a starting point for the development of social abilities, such as face processing^25,26^, and lack of social motivation might thus have cascade effects on other areas of social cognition. In ASD in particular, it has been argued that early deficits in social motivation and social reward responsiveness might have a long lasting impact on social skills^17,27,28^.

Recent developments in psychiatry (the Research Domain Criteria Framework) have emphasised the need to investigate variations in relevant biological traits across clinical and non-clinical populations^29–32^. Given its central role across conditions, social motivation has been identified as a relevant biological trait to investigate in a dimensional framework^33^. For instance, Parish-Morris *et al*.^34^ have shown using such a dimensional approach that individual differences in social attention is a better predictor of face processing skills than being diagnosed with ASD. Importantly however, the Research Domain Criteria Framework emphasizes that a given trait may have non-linear effects on behaviour, as is for example the case with the classic U-shaped curve relating stress and performance. In addition, variations in single traits do not necessarily have a uniform impact when they are taken in isolation and when they are combined in the context of psychiatric conditions^29–32^. Social anhedonia for instance, has a different effect on social cognition in patients with schizophrenia, patients with major depressive disorder and healthy controls^18,35^. In the case of ASD, stereotypical interests, anxiety, sensory peculiarities or number of co-frequent morbidities, such as anxiety, might also influence the way diminished social motivation alters individual behaviour^36,37^. It is therefore crucial to identify potential points of disjunction at which variations on a given traits affect cognitive functioning differently.

**Figure 1:**
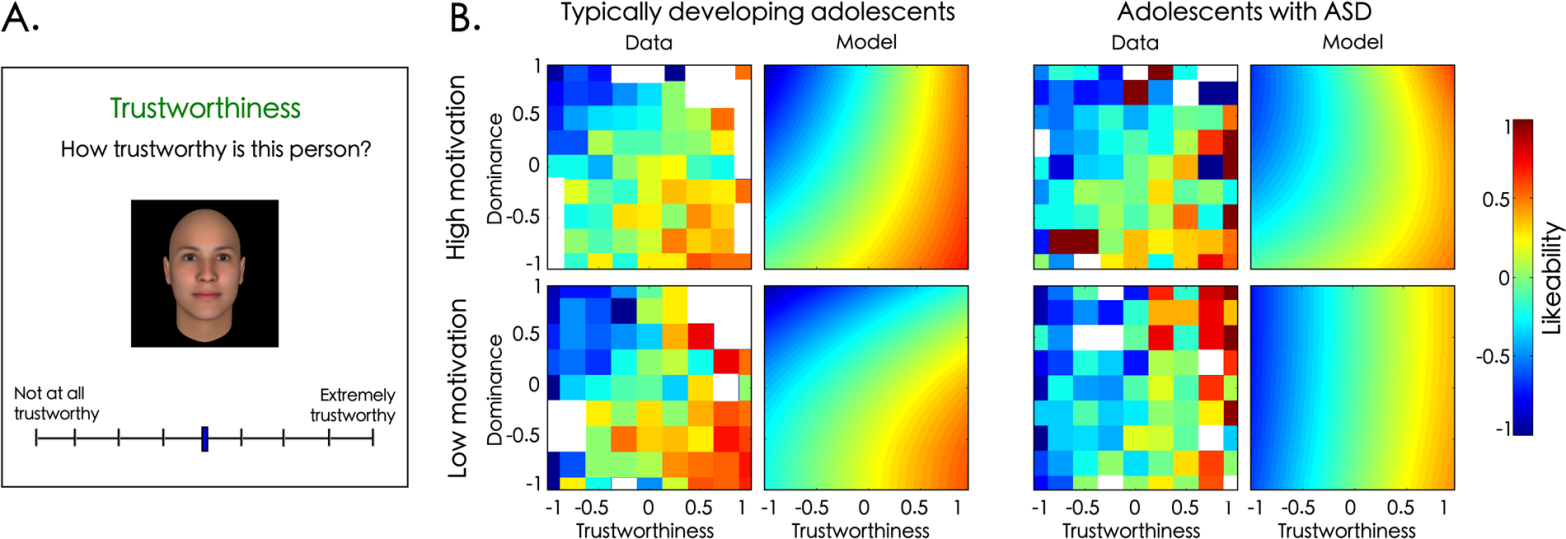
Social motivation has distinct effects on face evaluations in adolescents with and without ASD. (**A**) Example of an evaluation trial. Participants had to rate each avatar face^40^ by moving a cursor. (**B**) Likeability ratings as a function of trustworthiness (x axis) and dominance ratings (y axis) in typically developing adolescents (left) and adolescents with ASD (right). Rating intensity is represented on a scale ranging from blue for lower ratings to red for higher ratings. Pixelized figures correspond to averaged data in the initial study (data) for the most (upper row) and least (lower row) socially motivated participants (median split). Smoothed figures represent the predictions of the regression models ran separately on the two participant samples. While in typically developing adolescents, higher levels of social motivation are associated with an increase in the weight granted to trustworthiness, this is not the case in adolescents with ASD.

The aim of this paper is to apply the insights of dimensional approaches to the study of social motivation in ASD by testing whether social motivation has a uniform effect on individuals with and without ASD^31^. To investigate this question, we focused on face evaluation, which is key for social decision making^38^, and which is sensitive to variations in social motivation. Specifically, Safra *et al*. have shown that, when evaluating likeability in unknown faces, highly socially motivated adults are more influenced by perceived trustworthiness than by perceived dominance (Safra, L. *et al*., Submitted, 2018). Here, we asked 40 adolescent participants (20 typically developing (TD) and 20 with ASD) to rate faces on likeability using well-controlled stimuli varying parametrically in dominance and trustworthiness^39,40^ (Fig. 1A). More precisely, participants had to rate each of these faces on three traits (likeability, trustworthiness and dominance). We then measured participants’ self-reported pleasure in various activities using a validated questionnaire with items pertaining to social (e.g., “You accidentally overhear your teacher telling the principal what a terrific student you are”), physical (e.g., “You are cycling down the street very fast while still in good control of yourself”) or other sources of pleasure (e.g., “On a Saturday night, you stay up watching television as long as you want”)^41^. We then modelled participants’ likeability judgments as a combination of dominance and trustworthiness ratings using a mixed linear regression. Social motivation scores were also included in this model as a modulating variable. Our prediction was that higher levels of social motivation would increase the weight granted to trustworthiness during face evaluations in both TD and ASD populations.

## Results

### Cue processing in the TD and ASD groups

As our measure relied on the processing of facial features, we first checked that both groups were able to accurately detect and combine facial cues. Replicating previous findings^42,43^, TD adolescents successfully detected trustworthiness (*b* = 0.19 ± 0.03, *t*(579) = 13.95, *p* < 0.001; results are given in the standard form: mean ± 95% confidence intervals) and dominance cues (*b* = 0.16 ± 0.03, *t*(579) = 10.93, *p* < 0.001), giving higher trustworthiness/dominance ratings to avatars presenting higher levels of trustworthiness/dominance. Similarly, in line with previous studies^43–45^ adolescents with ASD successfully gave ratings that varied with the avatar’s level of trustworthiness and dominance (*b* = 0.18 ± 0.03, *t*(579) = 10.14, p < 0.001; *b* = 0.07 ± 0.05, *t*(579) = 2.81, *p* = 0.005).

Based on Oosterhof and Todorov^46^ and (Safra, L. *et al*., Submitted, 2018), we then reconstructed participants’ likeability two-dimensional space based on their ratings of dominance and trustworthiness. As can be seen in Fig. 1B, both adolescents with and without ASD combined perceived dominance and trustworthiness to form likeability judgments such that more trustworthy and less dominant faces were rated as more likeable in both groups (TD group: *b*_*Trustworthiness*_ = 0.47 ± 0.07, *t*(575) = 13.27, *p* < 0.001; *b*_*Dominance*_ = −0.32 ± 0.07, *t*(575) = −9.69, *p* < 0.001; ASD group: *b*_*Trustworthiness*_ = 0.52 ± 0.07, *t*(575) = 14.03, p < 0.001; *b*_*Dominance*_ = −0.07 ± 0.07, *t*(575) = −1.87, *p* = 0.062).

### Impact of social motivation in TD adolescents

We then examine whether, as in adults, social motivation increased the weight granted to trustworthiness for likeability evaluations in TD adolescents. As expected, social motivation increased the relative weight granted to trustworthiness to evaluate faces’ like ability (*b*_SocMot*Trust_ = 0.08 ± 0.08, *t*(570) = 2.06, *p* = 0.039; Fig. 1B). Importantly, this effect was still present after controlling for participants’ score in both non-social subscales (*b*_SocMot*Trust_ = 0.18 ± 0.11, *t*(560) = 3.24, *p* = 0.001). In addition, higher levels of social motivation were associated with lower sensitivity to high levels of dominance, such that highly socially motivated participants were more likely to approach faces rated as both highly dominant and highly submissive (*b*_SocMot*Dom_^2^ = 0.14 ± 0.12, *t*(570) = 2.41, *p* = 0.016; after controlling for non-social motivations: *b*_SocMot*Dom_^*2*^ = 0.13 ± 0.15, t(560) = 1.67, p = 0.098). A lower weight granted to dominance in highly socially motivated participants was also found as a trend, indicating that higher levels of social motivation is associated with a smaller importance given to perceived dominance when evaluating likeability in new faces (*b*_SocMot*Dom_ = 0.06 ± 0.07, *t*(570) = 1.71, *p* = 0.088; after controlling for non-social motivations: *b*_SocMot*Dom_ = 0.11 ± 0.10, t(560) = 2.29, p = 0.023; all other effects, *p* > 0.108). Given that social motivation was associated with an increase in the perceived intensity of trustworthiness (*b* = 0.03 ± 0.03, *t*(578) = 2.01, *p* = 0.045; other effects on cue detection: all *p*s > 0.104), we conducted a similar model using avatars’ objective dominance and trustworthiness as predictors. This analysis confirmed that social motivation was associated with a larger weight granted to trustworthiness for likeability evaluations (*b*_SocMot*Trust_ = 0.03 ± 0.01, *t*(570) = 3.42, *p* < 0.001; after controlling for non-social motivations: *b*_SocMot*Trust_ = 0.03 ± 0.02, *t*(560) = 3.35, *p* < 0.001; no other significant effect of social motivation: all *p*s > 0.124). As a conclusion, social motivation had a similar impact in adolescents as reported in adults (Safra, L. *et al*., Submitted, 2018).

### Effect of social motivation in ASD

We then tested whether social motivation had a uniform impact across populations by including Group (ASD *vs* TD) as a regressor in the model. The interaction between social motivation and the weight granted to trustworthiness was different in the two groups as revealed by a significant three-way interaction (*b*_Group*SocMot*Trust_ = −0.17 ± 0.15, *t*(1140) = −2.18, *p* = 0.029; no other difference in the effect of social motivation between the two groups was found: all *p*s > 0.127). Importantly, the result held after controlling for scores in the non-social subscales (*b*_Group*SocMot*Trust_ = −0.38 ± 0.21, *t*(1120) = −3.47, *p* < 0.001; no other significant interaction between Group and social motivation: all *p*s > 0.250). Finally, these findings were replicated using avatars’ levels of dominance and trustworthiness instead of participants’ subjective evaluations to predict likeability evaluations, suggesting that this difference was not due to differences in the way adolescents evaluate dominance and trustworthiness cues (*b*_Group*SocMot*Trust_ = −0.04 ± 0.03, t(1140) = −2.94, *p* = 0.003; after controlling for non-social motivations: *b*_Group*SocMot*Trust_ = −0.06 ± 0.04, t(1120) = −3.05, *p* = 0.002; all other interactions between Group and social motivation: all *p*s > 0.130).

In a secondary analysis, we focused on the ASD group alone and applied the exact same analyses on the ASD group as those previously conducted on the TD group. We found that social motivation was not associated with an increased weight granted to trustworthiness in the ASD group (*b*_SocMot*Trust_ = −0.05 ± 0.11, *t*(570) = −1.34, *p* = 0.182). In addition, adding the non-social subscores as control variables further revealed that social motivation was associated with a marginally significant decrease in the weight granted to trustworthiness for likeability evaluations in the ASD group (*b*_SocMot*Trust_ = −0.11 ± 0.09, *t*(560) = −1.87, *p* = 0.062). To summarize, our results thus suggest that social motivation does not have a uniform effect across the TD and the ASD groups.

In line with this finding, while the ASD group was less socially motivated than the TD group (*t*(38) = −2.43, *p* = 0.019), the comparison of these two groups did not match the difference between lowly and highly socially motivated adolescents without ASD. More precisely, compared to TD adolescents, adolescents with ASD perceived dominance cues as less intense (*b* = −0.09 ± 0.06, *t*(1158) = −3.04, *p* = 0.002; no other significant difference in cue detection: all *p*s > 0.250), and granted less weight to dominance for evaluating likeability (*b*_Group*Dom_ = 0.26 ± 0.10, *t*(1150) = 5.03, *p* < 0.001; no other significant effect of diagnosis: all *p*s > 0.113). Importantly, this effect was preserved when taking objective cues of dominance and trustworthiness as predictors (*b*_Group*Dom_ = 0.05 ± 0.02, *t*(1150) = 4.62, *p* < 0.001), which indicates that the weighting difference between the ASD and TD groups could not be explained by differences in explicit cue evaluation.

## Discussion

The aim of this study was to assess whether social motivation had a similar impact in adolescents with and without ASD. Previous work demonstrated that higher levels of social motivation increase the weight granted to trustworthiness cues during likeability evaluations (Safra, L. *et al*., Submitted, 2018). We replicated this effect in TD adolescents. However, social motivation had the opposite effect in the ASD group and was associated with a decrease in the weight granted to trustworthiness. This shows that social motivation can have contradictory effects in clinical and non-clinical populations. Given that ASD participants are overall less socially motivated than typically developing participants^19^, it would be tempting to construe autism as a simple case of extreme diminished social motivation and to use findings describing the effect of low social motivation in the general population as a guide to predict ASD cognition. Our results challenge this view, by revealing that atypicalities found in participants with ASD do not match the behavioural differences associated with diminished social motivation in a non-clinical sample: lowly socially motivated adolescents without ASD displayed a decreased sensitivity to trustworthiness, adolescents with ASD displayed a decreased sensitivity to dominance.

These findings have important implications for the understanding and investigation of ASD. First, our results suggest that the effect of social motivation uncovered in non-clinical populations cannot always be applied to ASD. Social disinterest in ASD may indeed be associated with emergent properties that cannot be derived by simply extrapolating the effects of mildly diminished social motivation. In addition, long-lasting difficulties in social interactions may have a retroactive action on social behaviour^47–50^. Finally, ASD are well-known for being associated with other conditions such as social anxiety and hyperactivity disorders^36,37^ that may interact with diminished social motivation. In this context, it is important to underline that findings obtained in subclinical or non-clinical populations should be applied to ASD with a great deal of caution.

Regarding the present study, we wish to underline two potential sources of noise. First, self-reports of social motivation might be biased differently in the ASD and the TD group: individuals with ASD indeed have difficulties reporting their own feelings and may lack insight^51–53^; conversely TD participants are more likely to be susceptible to social desirability effects^54^. Our results should thus be replicated using more objective measures of social motivation^10,55^. Second, it is widely recognised that autism should not be construed as a unique neuropsychological condition and that a composite view is needed in order to take into account the existence of distinct partial phenotypes in ASD^56,57^. It is thus possible that adolescents in our study belong to different subtypes of ASD that are characterized by different levels of social motivation deficits. Addressing these questions would require a much larger sample size than the one we had access to in the context of this study. Despite these caveats, we believe that our results are relevant for the understanding of social processing in ASD. In particular, we demonstrated that individuals with ASD are able to detect dominance and trustworthiness cues and to combine these cues to produce likeability evaluation (albeit differently from TD participants). These results are in line with a growing body of evidence showing preserved abilities to detect and to combine social cues in individuals with ASD^44,45,58–60^. In addition, our study also extends previous results on social cues combination by showing that individuals with ASD are not only able to modulate their perception of faces by integrating different social signals but also that they are able to create new social judgments by combining social cues. Indeed, the detection of dominance and trustworthiness cues and their combination are processed in distinct brain regions^61–63^.

Moreover, the impact of ASD on the way social cues are combined to produce likeability evaluations is particularly robust. Differences in likeability evaluations indeed remained while using both avatars’ objective and subjective levels of dominance and trustworthiness, which demonstrates an actual difference in dominance salience when producing likeability evaluations. Overall, our results thus suggest that individuals with ASD are able to detect and use dominance, but they place less weight on this trait compared to TD individuals. Why that might be the case is an empirical question in need of further investigation. Nevertheless, a number of experiments have revealed that more masculine individuals are less sensitive to dominance^64,65^. Therefore, our results may be in line with previous research showing an exaggerated male pattern of neural activation in ASD during face evaluation^66^. More widely, our results are relevant for dimensional approaches in psychiatry beyond the precise case of social motivation^30–32^. A growing body of research has indeed emphasized the importance of studying the impact of specific traits on behaviour by pooling clinical and non-clinical populations^29–32^. This approach has notably been applied by investigating biologically-relevant traits, such as reward anticipation across populations^67^; or by applying multi-dimensional diagnostic measures, such as the Autism Quotient, to sub- and non-clinical population^68^. However, our results suggest that findings obtained in non-clinical samples cannot always be directly mapped onto clinical populations. As suggested for the case of social motivation, it is indeed possible that variations in specific traits affect behaviour non-linearly to produce emergent behavioural peculiarities. Therefore, our findings are in favour of a strict application of the original Research Domain Criteria Framework recommendations emphasizing the importance of investigating biologically relevant traits across conditions and of identifying points of disjunction that may potentially give rise to non-linear effects of a given trait on behaviour^29–32^.

To summarise, our study replicates previous findings obtained in healthy adults showing that social motivation increases the weight granted to trustworthiness to produce likeability judgments. In contrast with our prediction however, social motivation did not have the same impact in ASD and in TD. Despite an overall diminished social motivation in the ASD group, the impact of autism was quite different than the simple effect of social motivation in TD adolescents. This result suggests that it may be misleading to construe social motivation in isolation and that it is vital to further understand how social motivation interacts with other dimensions of ASD.

## Material and Methods

### Participants

A minimum target of 20 TD adolescents and 20 adolescents with ASD was fixed *a priori* based on our past experience with social cognition experiments. In particular, a previous experiment conducted in our team on a sample of the same size^60^ successfully applied statistical and computational analyses to highlight differences and similarities in social stimuli processing between TD adolescents and adolescents with ASD. The exact number was determined by scheduling constraints. A final number 22 TD adolescents (6 females) and 22 adolescents with ASD (4 females), aged between 12 and 17 years old (TD: *M* = 13.70 ± 0.61; ASD: *M* = 14.45 ± 0.89; Table 1), participated in this study. The experiment was approved by INSERM and by the local research ethics committee (ClinicalTrials.gov Identifier: NCT02628808, Protocol Study ID: 2008-A00019-46) and was performed in accordance with the Declaration of Helsinki. The TD adolescents were recruited from a mainstream school and the adolescents with ASD were recruited from the University Hospital Robert Debré (Paris, France). The adolescents with ASD had received an official diagnosis of autism by an independent clinician according to the criteria of the Diagnostic and Statistical Manual for mental disorders-IV TR (DSM IV TR^69^). The Autism Diagnostic Interview Revised (ADI-R^70^) and the Autism Diagnostic Observational Schedule (ADOS^71^) were used to further assess the ASD group. The mean ADOS score for the ASD group was 13.59 ± 1.81 (Table 1). All participants with ASD had normal vision (Freiburg Visual Acuity and Contrast Test version 3.8.2^72^ adapted to the distance used in the experiment of 30 cm), no participant was on medication during the period of the study. Preliminary interviews confirmed that TD adolescent participants did not have any special needs or history of psychiatric illness or developmental delay and all of them had normal or corrected to normal vision.

**Table 1:** Descriptive statistics for age, gender and IQ and anxiety of the ASD and TD groups.

	TD (*N* = 20)	ASD (*N* = 20)	Statistics
Age	13.70 ± 0.61	14.00 ± 0.88	*t*(38) = 0.58, *p* > 0.250
Gender ratio	25% female	15% female	*χ*^*2*^(2, *N* = 42) = 0.01, *p* > 0.250
IQ	106.60 ± 6.14^a^	100.85 ± 10.13^b^	*t*(38) = −1.02, *p* > 0.250
STAI	13.80 ± 1.80	14.35 ± 1.96	*t*(38) = 0.43, *p* > 0.250
ADOS	n.a.	13.59 ± 1.81	
Social Anhedonia^c^	30.10 ± 1.74	34.10 ± 2.97	*t*(38) = −2.43, *p* = 0.019
Physical Anhedonia^c^	12.80 ± 0.91	13.10 ± 1.23	*t*(38) = −0.41, *p* = 0.684
Other Sources of Pleasure Anhedonia^c^	18.65 ± 2.06	21.15 ± 1.73	*t*(38) = −1.94, *p* = 0.059

^a^Full Wechsler Intelligence Scale for Children version IV (WISC IV^73^).

^b^Wechsler Abbreviated Scale of Intelligence (four subsets form^74^).

^c^Kazdin’s Pleasure Scale^41^.

Before testing, all parents and children provided their written informed consent to participate in the study. IQ was measured using the full Wechsler Intelligence Scale for Children version IV (WISC IV^73^; Mean = 103.40 ± 10.86; range: 70–148; Table 1) in adolescents with ASD and with the Wechsler Abbreviated Scale of Intelligence in TD adolescents due to time constraints (in the four subsets form as it has been shown to give the most representative score of the full IQ,^74^; range: 87–138; Table 1). Finally, at the end of the experiment, participants completed the Kazdin’s Pleasure Scale^41^, a self-rated questionnaire to assess their levels of anhedonia (see description below). In addition, trait anxiety was assessed using an abbreviated form of the State-Trait Anxiety Inventory (STAI^75,76^; Table 1).

## Materials and Design

### The pleasure scale

The Kazdin Pleasure Scale for Children (three subforms: social, physical and other;^41^; Table 1) was used to assess participants’ anhedonia levels. This scale is a validated self-rated instrument to measure anhedonia in both children with and without ASD^19^. It consists of 39 items pertaining to social (e.g., “You accidentally overhear your teacher telling the principal what a terrific student you are”), physical (e.g., “You are cycling down the street very fast while still in good control of yourself”) or other sources of pleasure (e.g., “On a Saturday night, you stay up watching television as long as you want”). Participants were asked to read each item out loud and to rate their feeling in the corresponding situation on a 3-point Likert scale (“Very happy” (scored 1), “Happy” (scored 2) or “Neither happy nor unhappy” (scored 3)). Therefore, higher scores to this scale indicates higher levels of anhedonia. The three scales were thus reverse-coded in order to reflect participants’ levels of motivation.

### The Face Evaluation Task

The experiment was programmed on ePrime (Psychology Software Tools, 2002) and lasted approximately 15 minutes. 30 faces varying parametrically on dominance and trustworthiness were generated using FaceGen 3.1 (http://www.facegen.com). Previous research has shown that these faces elicit dominance and trustworthiness judgments both at the explicit and the implicit level^39,40^. Following Oosterhof and Todorov’s methodology^46^, the questions bearing on the three traits of interest, i.e. trustworthiness, dominance or likeability, were presented in separate blocks. The three-block sequence and the sequence of trials within each block were randomized between blocks and between participants. Participants had to answer: ‘How [trait] is this person?’ using a cursor on a 9-point scale ranging from 1 ‘not at all [trait]’ to 9 ‘extremely [trait]’ (recoded from −1 to +1 for the analyses). Depending on the block, [trait] was replaced by ‘trustworthy’, ‘dominant’ or ‘likeable’. The face, the question and the scale appeared simultaneously after a 400 ms blank screen. Participants were instructed to follow their first impression and they were told that there was no right or wrong answer. The mouse was initially set to the middle of the screen in order to reinforce the salience of the positive and the negative sides of the scale. The name of the dimension was displayed in each trial (Fig. 1A).

### Procedure

Participants were tested individually in a quiet room. Participants were seated at a 30 cm distance from the laptop. They completed three separate blocks of the face evaluation task, each block consisted of the same 30 faces. Participants could rest between each block. Following completion of the 90-trial experiment, participants filled out the STAI and the Kazdin Pleasure scale with the experimenter.

### Data cleaning

2 TD participants and 2 ASD participants were excluded from the analysis for using only one side of the scales.

### Group differences

We first checked that the included participants of the ASD and TD groups were matched on age, gender, IQ and anxiety. The ASD and TD groups did not differ on any of these variables (Table 1). We then measured the difference between the ASD and TD groups in the different types of motivation. As in previous studies^19^, the ASD group was significantly less socially motivated than the TD group (*t*(38) = −2.43, *p* = 0.019) but did not differ in the two other types of motivation (Table 1). However, it is worth noting that contrary to Chevallier *et al*.^19^, social motivation did not significantly correlate with ADOS severity scores (coded as indicated in Gotham *et al*.^77^; *r* = −0.02 ± 0.44, *N* = 20, *t*(16) = 0.09, *p* > 0.250).

Finally, to test for possible differences between the ASD and the TD groups in the way participants performed the task, we ran several t-tests on scale use variables (i.e., ratings variance, number of different ratings, lower and higher ratings) for each scale. None of these values were significantly different between the two groups either for the dominance scale (all *p*s > 0.250), the trustworthiness scale (all *p*s > 0.250) or the likeability scale (all *p*s > 0.118).

### Data analysis

#### Cues decoding

To measure the influence of social motivation on participants’ ability to decode trustworthiness and dominance cues, we ran mixed linear regressions on trustworthiness and dominance ratings, taking avatars’ levels of trustworthiness/dominance as well as participants’ level of social motivation as regressors and participants’ ID as a random factor (R freeware, nlme package^78^).

#### Likeability evaluations

To investigate the impact of social motivation on the composition of likeability judgments, we ran a mixed linear regression on likeability evaluations, taking social motivation, ratings of dominance and ratings of trustworthiness as predictors and participants’ ID as a random factor. Following Todorov *et al*.^62^, this model included linear and quadratic interaction effects of perceived trustworthiness and perceived dominance as well as interaction terms between these two factors (R freeware, nlme package^78^). In addition, we also ran a similar model taking avatars’ objective levels of trustworthiness and dominance instead of participants’ ratings of trustworthiness and dominance (R freeware, nlme package^78^).

### Data availability statement

All the data and scripts for analyses are available at https://osf.io/rycu8/.
