# Author Correction: Distinct effects of social motivation on face evaluations in adolescents with and without autism

**DOI:** 10.1038/s41598-018-37593-5

**Published:** 2018-12-21

**Authors:** Lou Safra, Christina Ioannou, Frédérique Amsellem, Richard Delorme, Coralie Chevallier

**Affiliations:** 1 Laboratoire de Neurosciences Cognitives, Inserm unit 960, Département d’Etudes Cognitives, Ecole Normale Supérieure, PSL Research University, Paris, 75005 France; 2 Service de Psychiatrie de l’Enfant et de l’Adolescent, Hôpital Universitaire Robert Debré, Paris, 75019 France; 3 Génétique Humaine et Fonction Cognitive, Institut Pasteur, Paris, 75015 France

Correction to: *Scientific Reports* 10.1038/s41598-018-28514-7, published online 13 July 2018

The Acknowledgements section in this Article is incomplete.

“This study was supported by the Institut d’Études Cognitives (ANR-10-LABX-0087 IEC and ANR-10-IDEX-0001-02 PSL*). We are also grateful to Dr. Bahador Bahrami for providing us the stimuli.”

should read:

“This study was supported by Frontiers in Cognition (FrontCog, ANR-17-EURE-0017). We are also grateful to Dr. Bahador Bahrami for providing us the stimuli and to Raphael Delage for his valuable help in data collection.”

